# *Xkr8* Modulates Bipolar Cell Number in the Mouse Retina

**DOI:** 10.3389/fnins.2018.00876

**Published:** 2018-12-03

**Authors:** Amanda G. Kautzman, Patrick W. Keeley, Caroline R. Ackley, Stephanie Leong, Irene E. Whitney, Benjamin E. Reese

**Affiliations:** ^1^Neuroscience Research Institute, University of California, Santa Barbara, Santa Barbara, CA, United States; ^2^Department of Psychological and Brain Sciences, University of California, Santa Barbara, Santa Barbara, CA, United States; ^3^Department of Cellular, Molecular and Developmental Biology, University of California, Santa Barbara, Santa Barbara, CA, United States

**Keywords:** recombinant inbred strain, gene variant, *cis*-eQTL, electroporation, cell death

## Abstract

The present study interrogated a quantitative trait locus (QTL) on Chr 4 associated with the population sizes of two types of bipolar cell in the mouse retina. This locus was identified by quantifying the number of rod bipolar cells and Type 2 cone bipolar cells across a panel of recombinant inbred (RI) strains of mice derived from two inbred laboratory strains, C57BL/6J (B6/J) and A/J, and mapping a proportion of that variation in cell number, for each cell type, to this shared locus. There, we identified the candidate gene *X Kell blood group precursor related family member 8 homolog* (*Xkr8*). While *Xkr8* has no documented role in the retina, we localize robust expression in the mature retina via *in situ* hybridization, confirm its developmental presence via immunolabeling, and show that it is differentially regulated during the postnatal period between the B6/J and A/J strains using qPCR. Microarray analysis, derived from whole eye mRNA from the entire RI strain set, demonstrates significant negative correlation of *Xkr8* expression with the number of each of these two types of bipolar cells, and the variation in *Xkr8* expression across the strains maps a *cis*-eQTL, implicating a regulatory variant discriminating the parental genomes. *Xkr8* plasmid electroporation during development yielded a reduction in the number of bipolar cells in the retina, while sequence analysis of *Xkr8* in the two parental strain genomes identified a structural variant in the 3^′^ UTR that may disrupt mRNA stability, and two SNPs in the promoter that create transcription factor binding sites. We propose that *Xkr8*, via its participation in mediating cell death, plays a role in the specification of bipolar cell number in the retina.

## Introduction

The sizes of various neuronal populations in the mouse retina have been shown to exhibit little inter-individual variation with a strain, yet there is substantial variation between strains (Keeley et al., 2014b). Such between-strain variation has been mapped to specific genomic loci, where variant genes modulate biological processes affecting fate determination, differentiation, and cell survival (Whitney et al., 2009, 2011a,b). Curiously, the variation in cell number exhibits little co-variation between different cell types, indicating that the final number of cells, for each type, is not coordinated by any single gene, for example, by affecting retinal proliferation. Rather, the variation in the final number of each cell type is largely modulated independent of one another, regardless of whether they are synaptically connected or developmentally related (Keeley et al., 2014b). For instance, the numbers of rod photoreceptors and rod bipolar cells are not significantly correlated across 26 recombinant inbred (RI) strains of mice, and nor are the numbers of cone photoreceptors with either the Type 2, the Type 3b, or the Type 4 cone bipolar cells. Likewise, the numbers of rod and cone photoreceptors show no significant correlation, and the three types of cone bipolar cells are also not significantly correlated with each other (Keeley et al., 2014b).

The exception to this is a significant co-variation between rod bipolar cells (RBCs) and Type 2 cone bipolar cells (CBC2s). Some of this variation in each of these cell types mapped to the same genomic locus on Chromosome (Chr) 4, where we scrutinized this particular interval for promising candidate genes that control bipolar cell number. In this study, we identified one such candidate gene at this locus, *Xkr8*, and describe how variation in its expression across these RI strains correlates with both RBC number and CBC2 number. We show significant differences in *Xkr8* retinal expression between C57BL/6J and A/J mouse strains during the postnatal period, and demonstrate that manipulating *Xkr8* expression shortly after birth modulates the frequency of bipolar cells. As *Xkr8* promotes phosphatidylserine exposure in apoptotic cells (Suzuki et al., 2013, 2014), a *cis*-regulatory variant in *Xkr8* may ultimately modulate bipolar cell number by affecting naturally occurring cell death.

## Materials and Methods

### Mice

RBCs and CBC2s were counted in C57BL/6J (hereafter B6/J) and A/J mice, and in 26 genetically distinct recombinant inbred (RI) strains derived from them comprising the AXB/BXA strain set. The cell count data from these mice have previously been reported in an on-line appendix (Keeley et al., 2014b), while a more extensive study has recently reported the cell counts and quantitative trait locus (QTL) analysis for the RBC population (Kautzman et al., 2018); the methodology associated with these analyses is briefly recapitulated below. Eyes from *Xkr8* knockout mice (KO) and heterozygous (Het) littermate control mice (wild type littermate controls were not available) were provided by the laboratory of Dr. Shigekazu Nagata at Osaka University in Japan (Suzuki et al., 2013). Electroporation experiments were conducted on CD1 mice originally obtained from Charles River Laboratories (Crl:CD1, #022), and subsequently bred in the UCSB Animal Resource Center. B6/J and A/J mice were also bred in-house, and used for *in situ* hybridization, immunofluorescence and qPCR studies. All experiments were carried out under authorization by the Institutional Animal Care and Use Committee at UCSB, and in accord with the NIH *Guide for the Care and Use of Laboratory Animals.*

### Tissue Preparation

In order to determine the total number of RBCs and CBC2s in mice of the AXB/BXA RI strain set, 4–8 week old mice were given a lethal injection of sodium pentobarbital (120 mg/kg, i.p.; Virbax, Fort Worth, TX, United States) and subsequently perfused intracardially with 2 ml of 0.9% saline followed by ∼75 ml of 4% paraformaldehyde in 0.1 M sodium phosphate buffer (PB, pH 7.2). Eyes were removed and left in the same fixative for an additional 15 min at room temperature. Eyes from all electroporated mice, as well as those from postnatal mice less than 2 weeks of age, were immersed in 4% paraformaldehyde for 30 min at room temperature in lieu of perfusion. Following fixation, retinas were dissected from the eyes taking care to maintain the entirety of the retina, and four radially-oriented relieving cuts (extending half-way to the optic nerve head) were made to enable the retina to lie flat. The vitreous was gently brushed from the inner surface of the retina, and the wholemount then rinsed in PB. Alternatively, dissected retinas were embedded flat in 5% agarose and sectioned radially on a Vibratome at 150 μm.

### Immunofluorescence

All retinal wholemounts and sections were incubated in 5% normal donkey serum for 3 h, washed, and incubated in primary antibody for 72 h. For cell number quantification, retinas were immunolabeled with either a polyclonal rabbit antibody to protein kinase C (PKC, 1:10,000, Cambio; #CA1024) to label RBCs or a mouse monoclonal antibody to synaptotagmin 2 to label CBC2s (SYT2, 1:100; Zebrafish International Resource Center; #ZDB-ATB-081002-25). GFP-positive cells in electroporated retinas were detected using a rabbit polyclonal antibody to green fluorescent protein (GFP, 1:1,000, Molecular Probes; #A21311). Radial sections were also co-stained with Hoechst 33342 (1:1,000, Invitrogen; #H3570) to label cellular nuclei. To characterize the distribution and co-localization of XKR8, an affinity-purified rabbit polyclonal antibody to XKR8 (1:100, Sigma; #HPA053529) was used in adult and developing retinal sections. Adult retinal sections were also double-labeled with the above mouse monoclonal antibody to SYT2, or a mouse monoclonal antibody to PKC (1:500, Millipore; #05-983). Retinas were then washed and incubated in secondary antibodies of donkey anti-rabbit, donkey anti-goat, or donkey anti-mouse IgG conjugated to Cy2 or Cy3 (1:200, Jackson ImmunoResearch Labs) overnight. All washes were completed three times in phosphate-buffered saline (PBS) for 5 min each, and all steps were completed with agitation at 4°C. Primary and secondary antibody solutions were prepared in PBS with 1% Triton X-100.

Since labeling with the XKR8 antibody has never been described in the mouse retina, two control experiments were performed. First, the primary antibody was pre-absorbed with its corresponding antigen peptide (10 μg peptide:1 μg antibody; Atlas Antibodies, #APrEST89697) for 30 min at room temperature to block all specific staining of the antibody to XKR8 protein. Second, the primary antibody was omitted from some sections to determine if any non-specific labeling from the secondary antibodies was present. These controls were performed on tissue collected from the same B6/J animal and run concurrently with the normal protocol.

### Quantification of Cell Number

**FIGURE 1:**
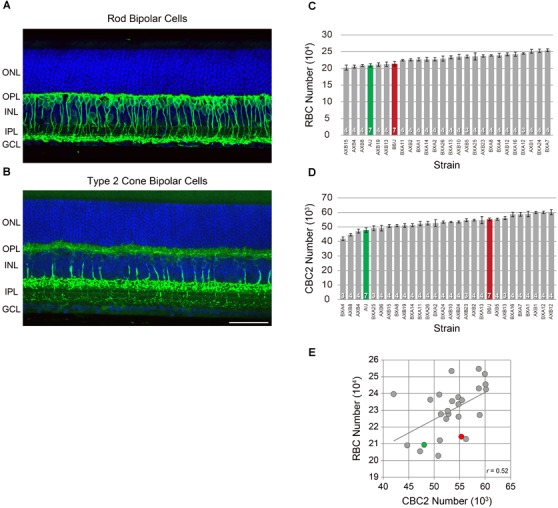
RBCs were labeled using antibodies to PKC **(A)**, while CBC2s were labeled using antibodies to SYT2 **(B)**. Each antibody labeled the somata, dendritic arbors, axons, and terminals. Both are shown here in retinal sections taken from adult B6/J mouse retinas, but were quantified in retinal wholemounts by counting their labeled axons. RBC number **(C)** and CBC2 number **(D)** each showed conspicuous variation across the RI strains of the AXB/BXA strain set (gray) in excess of the difference between the two parental strains B6/J (red) and A/J (green). n = the number of eyes sampled from different mice in each strain. **(E)** Cell number co-varied across the strains. Pearson’s correlation coefficient was statistically significant (*p* < 0.05). Scale bar = 50 μm.

Immunolabeled retinas from the parental inbred laboratory strains B6/J and A/J, and from the genetically distinct RI strains from the AXB/BXA strain set were mounted flat in 0.1 M phosphate buffer with the ganglion cell layer oriented facing the coverglass, and examined using an Olympus BH2 fluorescence microscope coupled via a Sony video camera to a computer. Four fields, each positioned at a mid-location between the retinal circumference and optic nerve head, were sampled, with one in each quadrant of the retina and the areal size of each sampling field being 0.016 mm^2^. Every PKC immunopositive axon (for RBCs), or SYT2 immunopositive axon (for CBC2s) coursing through the inner nuclear layer (INL) into the inner plexiform layer (IPL) within the sampling field was counted by eye. The area of each wholemount was measured using Bioquant Nova Prime software (R&M Biometrics), and retinal area was multiplied by the average density of bipolar cells across the four fields in order to estimate total bipolar cell number for each population. Three to four mice were sampled in each RI strain, while seven mice were sampled in each of the parental B6/J and A/J strains. Typically three strains of mice were processed in a batch, with all mice being individually coded and randomly intermingled, so that all counting was conducted blind to strain. Individual fields from retinal sections were imaged using an Olympus Fluoview 1000 laser scanning confocal microscope with a 40× objective, in which images were collected at 1 μm intervals and prepared as Z-stack reconstructions. Counts of RBCs were not obtained from 2 of the RI strains, so the correlation analysis shown in Figure 1E is derived from 24 of the 26 RI strains.

### QTL Mapping and Interval Analysis

**FIGURE 2:**
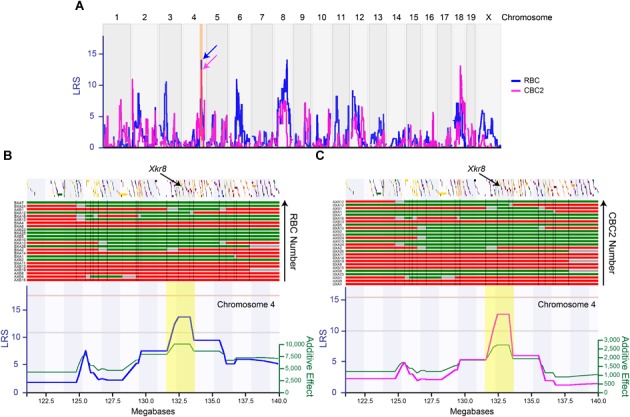
The variation in RBC number (blue trace) and CBC2 number (magenta trace) each mapped to multiple genomic loci **(A)**, including one shared locus on Chr 4 (arrows in **A**). The expanded map of a distal portion of Chr 4 (120–140 Mb, highlighted in orange in **A**) is shown below (in **B,C**), with the LRS plots now shown separately for each cell type, with RBCs on the left **(B)** and CBC2s on the right **(C)**. The position of all genes is indicated across the top, along with the B6/J versus A/J haplotypes across the RI strains (red and green bars, respectively, ordered from bottom to top according to cell number in ascending order). In addition, the additive effect of each *A* allele (green trace) is shown beneath each LRS trace. The interval interrogated at the Chr 4 locus extended from 131.5 to 133.5 Mb (highlighted in yellow in **B**,**C**), and encompassed 55 genes. Gray regions indicate undetermined haplotype blocks. The position of *Xkr8* is indicated, at 132.85 Mb. Horizontal pink and gray lines in both **(B,C)** indicate the significant and suggestive LRS thresholds determined by permutation testing.

Simple interval mapping was conducted with the aid of the mapping module in GeneNetwork^1^. The original phenotype data (RBC total number and CBC2 total number) have been entered into the AXB/BXA Phenotypes database in GeneNetwork as accession record IDs #10202 and #10181, respectively. Both datasets have been published in Table form in an appendix from a study comparing QTLs across 12 different retinal cell types (Keeley et al., 2014b), and a fuller account of the RBC dataset has been published (Kautzman et al., 2018). Permutation testing of the RI strain data was conducted to determine the probability of achieving likelihood ratio statistics (LRS scores) by chance. Thresholds for suggestive (*p* = 0.67) and significant (*p* = 0.05) LRS scores are indicated by the horizontal lines in Figures 2B,C, 5C. The right Y axis in each of these figures indicates the additive effect of each parental allele at each locus; because the RI strains (like the originating parental strains) used in the present analysis are all homozygous at each locus, this value is doubled to determine the additive effect of each QTL upon cell number or transcript levels. The Mouse Genome Assembly from 2011 (GRCm38/mm10) was used to define megabase positions of intervals.

The Sanger Institute Mouse Genomes Project database (Keane et al., 2011; Yalcin et al., 2011) was used to identify potential candidate genes with variants between the B6/J and A/J strains, looking specifically for variants in putative regulatory regions, being defined as those falling within the 5^′^ or 3^′^ untranslated regions (UTR) or within adjacent upstream or downstream sequences, which may alter gene expression levels, as well as for non-synonymous mutations that may alter protein function. The Mouse Retina SAGE library (Blackshaw et al., 2004) and B6/J microarray expression analysis (Freeman et al., 2011) were used to determine the expression of those genes housing parental genetic variants in the developing and adult retina, respectively. A microarray database derived from whole eye mRNA from each of the 26 RI strains of the AXB/BXA strain set and the parental strains (GeneNetwork AXB/BXA Whole Eye Expression Microarray Database, “*Eye AXBXA Illumina V6.2(Oct08) RankInv Beta Database*”) was also examined to identify genes for which expression across the strains correlated with variation in cell number, and for which expression mapped a potential *cis*-regulatory expression QTL (*cis*-eQTL). Our strategy for prioritizing genes is described in greater detail elsewhere (Kautzman et al., 2018); for genes that met these two latter criteria, they were investigated in further detail for known functions described in the literature. The full list of candidates at this Chr 4 locus are provided in a supplemental table in that former study. The present study focused exclusively on *Xkr8*, for reasons outlined in Section “Results.”

### Quantification of Retinal Thickness and Cell Density in *Xkr8* KO Mice

To quantify retinal thickness in the *Xkr8* KO and heterozygous littermate mice, two 40× magnification images were taken per retinal section, each ∼0.7 mm from the optic nerve head. Using Fiji software^2^, the Hoechst-labeled images were divided into 10 equal portions and the thickness of each retinal layer was measured [outer nuclear layer (ONL); outer plexiform layer (OPL); inner nuclear layer (INL); inner plexiform layer (IPL); ganglion cell layer (GCL)]. All measurements were averaged to yield mean thickness measurements for each layer, and summed to determine total retinal thickness. RBC and CBC2 numbers were counted from immunolabeled retinal sections at the same eccentricity, collecting four sampled fields per retina. Sampled fields were 11 μm-thick Z-stack projections, being 317 μm in length. Each of the four images from the seven eyes was assigned a random number, and the immunolabeled bipolar cells within the images were then counted blind to genotype. Animal averages were determined for each cell type, and expressed as cells per linear mm.

### *In situ* Hybridization

RNA *in situ* hybridization was used to visualize the spatial expression pattern of *Xkr8* in the mature B6/J mouse retina (2 months of age) according to an established protocol (Palop et al., 2011). Antisense and sense riboprobes specific to *Xkr8* were created using PCR and *in vitro* transcription (Ghafoory et al., 2012), as follows: primers were designed to specifically amplify a 482 bp sequence of *Xkr8* mRNA (forward primer: CATGTGGCCTTAGACGTGGT; reverse primer: CTGATGCCAAACCACTGGTAG) and were coupled to adaptor sequences containing the T3 (forward primer: GAAATAATTAACCCTCACTAAAGGG) or T7 (reverse primer: GAAATTAATACGACTCACTATAGGG) RNA polymerase promoter sites. cDNA generated from adult B6/J retinal tissue (iScript Advanced cDNA synthesis kit; BioRad, Hercules, CA, United States; #1725037) was used as a template and the resulting PCR product was purified. This product was subsequently used for *in vitro* transcription reactions with digoxigenin-labeled nucleotides (Roche, Switzerland; #11277073910) and either the T7 or T3 RNA polymerase (Promega, Madison, WI, United States; #P2075 and #P2083) was used to generate antisense and sense riboprobes, respectively.

Adult B6/J eyes were immersion-fixed as described previously, with care taken to avoid RNAse contamination of all solutions, tools, and working surfaces. Dissected retinas were then cut into 20 μm thick sections on a Microm HM560 cryostat (Thermo Scientific, Waltham, MA, United States) and adhered to precleaned Superfrost Plus glass slides (Fisher Scientific, Pittsburgh, PA, United States; #12-550-15). Sections were digested and acetylated in preparation for riboprobe hybridization as described by Palop et al. (2011), and then incubated with the purified *Xkr8* riboprobes (500 ng probe/mL at 65°C, overnight) to hybridize with the native *Xkr8* mRNA. A sheep antibody to digoxigenin conjugated to alkaline phosphatase (Roche; Indianapolis, IN, United States #11093274910) was subsequently used to detect the digoxigenin-labeled probes (1:5,000 at 4°C, overnight). Alkaline phosphatase activity was detected by developing the sections in a solution of NBT/BCIP (Roche; #11681451001) until a purple/blue precipitate could be observed, after which the sections were dehydrated in ethanol, cleared using Neo-Clear (Millipore, Billerica, MA, United States; #109843), and mounted with a coverslip. Slides were imaged on an Olympus BHS fluorescence microscope equipped with a video camera, using a 20× objective.

### Real Time-PCR (qPCR)

Retinas from A/J and B6/J animals at P1, P5, and P10 were collected in RNase-free conditions (3 separate litters, 6–8 retinas per litter). The RNAeasy Plus Mini Kit (Qiagen, Hilden, Germany) was used to extract RNA and single stranded cDNA was made via the iScript cDNA synthesis kit (Bio-Rad, Hercules, CA, United States). The Sso Advanced Universal SYBR Green Supermix (Bio-Rad, Hercules, CA, United States) was used to measure *Xkr8* expression levels on the Bio-Rad CFX Touch Real-Time PCR system. We empirically determined the proper annealing temperature for each primer set and ran all sets in triplicate on separate plates, using the median of the technical triplicates. Values were corrected for primer efficiency, melting temperature, and product size and *Xkr8* expression was normalized to the expression of housekeeping genes β-2 microglobulin (*β2m*), TATA-binding protein (*Tbp*), and Glyceraldehyde 3-phosphate dehydrogenase (*Gapdh*). Table 1 lists the primer sequences, product size, annealing temperature, and calculated efficiency for *Xkr8* and the three housekeeping gene primer sets.

**Table 1:** qPCR primer sequences, product sizes, annealing temperatures and calculated efficiencies for *Xkr8* and three housekeeping genes.

Gene	Forward primer (5^′^–3^′^)	Reverse primer (5^′^–3^′^)	Product size (bp)	Annealing temperature (°C)	Calculated efficiency (%)
*Xkr8*	CGGCTCAGAAGATCACTTCC	ACCCATTGTTCCAGTGAAGG	105	63.0	94
*Gapdh*	AACTTTGGCATTGTGGAAGG	GGATGCAGGGATGATGTTCT	132	60.0	98
*TBP*	CTCAGTTACAGGTGGTGGCAGCA	CAGCACAGAGCAAGCAACTC	120	61.5	94
*β2M*	GAGCCCAAGACCGTCTACTG	GCTATTTCTTTCTGCGTGCAT	134	61.5	97

### *In vivo* Electroporation

An *Xkr8* coding sequence (*Xkr8* #: 381560; GE Healthcare Dharmacon, Lafayette, CO, United States) was inserted into the multiple cloning site of the pCAGIG vector (Addgene, Cambridge, MA, United States; #11159) to make the *Xkr8*-expression plasmid. The pCAGIG plasmid alone was used as a control vector, and in both cases, included a downstream IRES-*GFP* sequence. P2 animals were anesthetized via hypothermia and a 27.5 gauge needle was used to sever the presumptive eyelid following cessation of movement. This needle was then used to puncture a small hole through the sclera. A 32 gauge Hamilton syringe was inserted and 0.6–0.8 μl of either *Xkr8* or control plasmid DNA was injected into the sub-retinal space of one eye. Pups were then delivered five 50 ms square wave pulses with 950 ms intervals of 80 V via paddles placed on either side of the head (BTX ECM 830, Holliston, MA, United States). Pups recovered in a bowl floating in a 42°C water bath and were returned to their dams upon vocalization and ambulation. At P21, mice were euthanized, and retinas were subsequently dissected flat, embedded, and sectioned radially, as described above. Sections with GFP-positive cells were subsequently enhanced by immunostaining with an anti-GFP antibody. The frequency of GFP-positive cells in the outer half of the INL (where bipolar cells reside), as a proportion of all GFP-positive cells (which were found only in the ONL and INL), was determined for each eye, derived from sampling fields containing GFP-positive cells across multiple sections. Twenty-five mice were successfully electroporated, yielding 10 *Xkr8* eyes and 15 control eyes for quantitation. All quantification was conducted blind to treatment, by coding individual images from treated and control retinas and then interleaving them randomly before counting.

### Sequencing the *Xkr8* Structural Variant

Genomic DNA from A/J and B6/J animals was extracted from tail tissue, and primers flanking the suspected structural variant were used to amply the genomic region of interest using PCR (forward primer: GGACTCCAGATGCCTCTGTAGCAG; reverse primer: GGCTCAGAAGATCACTTCCCGGAG). Purified fragments were then sequenced using a commercial Sanger sequencing service (Eton Bioscience, San Diego, CA, United States), and aligned to the reference genome (GRCm38/mm10).

### Statistical Analysis

A Student’s two-tailed *t*-test was used to determine statistical significance for electroporation experiments and for comparisons between *Xkr8* KO and heterozygous mice. The relationship between cell number and gene expression was calculated using Pearson’s correlation coefficient *r*. Results from qPCR experiments were tested for statistical significance between strains of mice, across developmental ages, and for an interaction between stain and age, using a two-way ANOVA followed by *post hoc* Tukey tests. A *p*-value ≤ 0.05 was used for determining statistical significance.

## Results

Like other types of retinal neurons, rod bipolar cells (RBCs; Figure 1A) and cone bipolar cells (CBC2s; Figure 1B) show conspicuous variability in their total number across this panel of recombinant inbred (RI) strains of mice. As previously reported, RBC number varies from a low of ∼202,000 cells to a high of ∼254,000 cells, while CBC2 number shows an even greater variation across the strains, from a low of ∼42,000 to a high of ∼61,000 cells (Keeley et al., 2014b; Kautzman et al., 2018). The variation in each cell type is graded across the strain set (Figures 1C,D), indicating that each is a complex trait that is controlled by multiple variant genes discriminating the parental strains. Note as well that the variation across the RI strains is more extensive than the difference between the parental strains themselves. Despite such pronounced variation in total cell number across the strains, the variation within any strain was meager, showing an average coefficient of variation of 0.05 across the strains, for each cell type (Keeley et al., 2014b). Curiously, these two cell types showed significant co-variation in their number (Figure 1E; *p* = 4.22 × 10^-3^), that was not present between most other pairs of cell types assessed across this family of RI strains (Keeley et al., 2014b).

The variation in cell number, for each cell type, mapped to multiple suggestive genomic loci, including QTL on Chrs 4, 6, and 8 for RBC number, and on Chrs 2, 4, and 18 for CBC2 number (blue and magenta traces, respectively, in Figure 2A). Of particular note, the locus on Chr 4 was shared for both cell types (arrows in Figure 2A), where the presence of *A* alleles at this locus was associated with an increase in the number of each cell type (Figures 2B,C). The additive effect of carrying the *A* haplotype at this locus for the RBC population was 19,294 cells (Kautzman et al., 2018), while for the CBC2 population it was 5,571 cells. In each case, the magnitude of the QTL effect exceeded 29% of the total variation in each population observed across the strains (Figures 1C,D).

The interval at this genomic locus extended from 131.5 to 133.5 Mb (highlighted in yellow in Figures 2B,C), and contained a total of 55 genes. We subsequently interrogated every one of these, winnowing down this list by eliminating those that had no known variants discriminating the two parental genomes, as well as those for which there is no documented retinal expression, while elevating for further consideration those suspected to play a role in biological processes affecting cell number and for which expression data correlated with cell number across the RI strain set. We focus the remainder of this paper on one particular gene at this locus on Chr 4, *Xkr8*.

**FIGURE 3:**
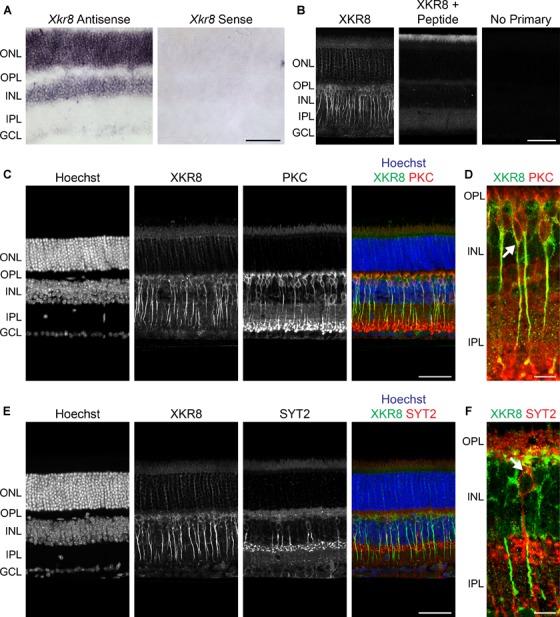
**(A)** *Xkr8* mRNA was localized in the mature B6/J mouse retina using *in situ* hybridization, evincing conspicuous expression in the ONL and INL, and weaker expression in the GCL (left). No signal was detected using the control (sense) riboprobe (right). **(B)** XKR8 immunofluorescence in the mature B6/J retina, showing prominent labeling of bipolar cell somata and axons, as well as lighter labeling of cells in the ONL and GCL (left). Positive control sections, in which the primary antibody was pre-absorbed with XKR8 peptide sequence (middle), and negative control sections, in which the primary antibody was omitted (right), showed abolition of labeling. **(C)** XKR8 labeling of bipolar cells includes the population of RBCs, though excluding their terminals in the IPL. **(D)** Higher magnification image showing XKR8 localized to RBC somata (e.g., arrow) and axons. Note as well-occasional XKR8-positive bipolar cells that are not RBCs (i.e., are PKC-negative). **(E)** XKR8 labeling is notably lighter in the membrane of CBC2 cells. **(F)** Higher magnification image showing XKR8 labeling in SYT2+ soma (arrow). Scale bar = 50 μm for **(A–E)**; = 10 μm for **(D,F)**.

The gene *Xkr8*, or *X Kell blood group precursor related family member 8 homolog*, belongs to a large family of evolutionarily conserved XK proteins, the functions of which are not well-understood (Calenda et al., 2006). Interestingly, XKR8 has recently been shown to play a role in promoting phosphatidylserine exposure on the surface of dying cells, facilitating phagocytosis (Suzuki et al., 2013, 2014; Sivagnanam et al., 2017). While there is no recognized role for *Xkr8* in the retina, by using *in situ* hybridization, we confirmed expression in the ONL, INL, and GCL in adult B6/J mouse retinal tissue (Figure 3A, left). Sense strand riboprobes exhibited no signal throughout the retina (Figure 3A, right).

We used an XKR8 antibody to immunolabel mature B6/J retinal sections, showing conspicuous membranous labeling of cells in the INL, and additional labeling of cells in the ONL and GCL (Figure 3B, left). Negative control sections, in which the primary antibody was eliminated, showed no labeling whatsoever (Figure 3B, right), while positive control sections, in which the primary antibody was pre-absorbed with the peptide sequence used in the generation of the antibody, showed a loss of all XKR8 labeling (Figure 3B, middle; compare with left panel). Double-labeling with antibodies to either PKC or SYT2 (to label the RBCs and CBC2s, respectively), confirmed strong labeling of RBC somata and axons, though diminishing at the level of the terminals (Figures 3C,D), while CBC2s, by contrast, showed only fainter labeling restricted to the somata (Figures 3E,F).

**FIGURE 4:**
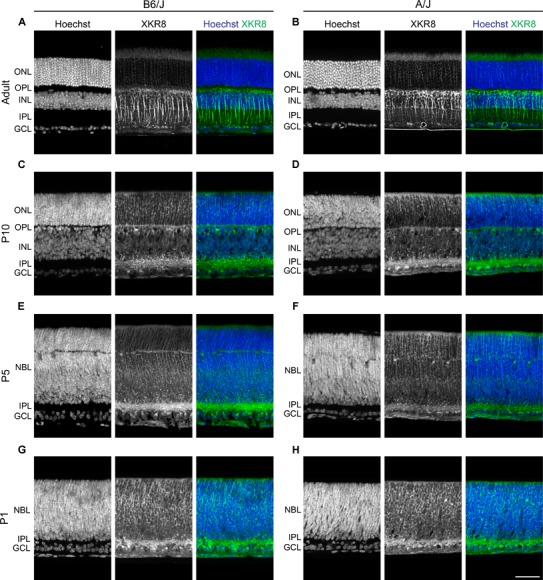
**(A,B)** Strain comparison of XKR8 immunofluorescence in the mature retina, showing a comparable pattern of bipolar cell labeling in B6/J (left) and A/J (right) retinal sections. **(C,D)** At P10, labeling of newly differentiating bipolar cells was detected, as well as labeling in the ONL and amongst amacrine and ganglion cells and their processes in the IPL. **(E–H)** At P5 and P1, labeling was present in the inner retina and within the neuroblast layer. Scale bar = 50 μm.

This pattern of labeling was comparable between the B6/J and A/J strains in the mature retina (Figures 4A,B). Labeling at P10 was similar to that in maturity, although there was greater amacrine cell and IPL labeling at this stage (Figures 4C,D). Labeling was present at P5 and P1 (Figures 4E–H), being most notably associated with the inner retina including the IPL, though including cells throughout the neuroblast layer at P1 (Figures 4G,H).

**FIGURE 5:**
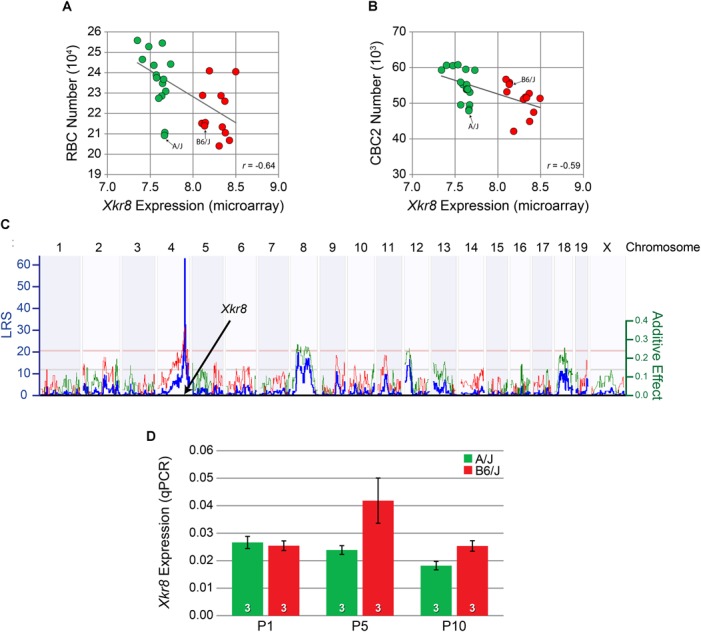
*Xkr8* expression was negatively correlated with both RBC number **(A)** and CBC2 number **(B)** across the RI strain set. Those strains carrying the *B* haplotype at the *Xkr8* locus (red) all had lower transcript levels relative to strains carrying the *A* haplotype (green). Expression in each of the parental B6/J and A/J strains is also indicated. **(C)** The variation in expression of *Xkr8* across the RI strains mapped to the same genomic locus for bipolar cell number, indicating the presence of a variant in *Xkr8* affecting gene expression (i.e., a *cis*-eQTL). **(D)** qPCR for *Xkr8* transcript levels showed significantly greater developmental expression in the B6/J strain relative to the A/J strain (*p* < 0.05), being most conspicuously greater at P5. Retinal mRNA was pooled from individual eyes in each litter, at each time-point. n = the number of litters per strain examined at each age.

Microarray expression data derived from adult whole eye mRNA from each of the RI strains of the AXB/BXA strain set showed a greater than twofold variation in *Xkr8* expression across the strains. That variation in expression showed a significant negative correlation with both RBC number (*p =* 3.25 × 10^-4^) (Figure 5A) as well as CBC2 number (*p =* 6.86 × 10^-4^) (Figure 5B). Furthermore, the variation in *Xkr8* expression across the strains itself mapped a significant expression QTL (eQTL) at the same Chr 4 locus (LRS = 62.5), i.e., a genomic locus where a sequence variant must contribute to the variation in gene expression itself, that is, a *cis*-eQTL (Figure 5C). Indeed, every strain with the *B* haplotype at this locus had greater levels of expression relative to those with the *A* haplotype (Figures 5A,B). *Xkr8* sequence in the DBA/2J genome is identical to that for A/J, and consequently differs from B6/J in the same nucleotides, and *Xkr8* expression derived from adult retinal mRNA from each of the RI strains of the BXD strain set (originating from the parental strains B6/J and DBA/2J) similarly maps a significant *cis*-eQTL (GeneNetwork BXD Retina mRNA, “*Full HEI Retina Illumina V6.2 (Apr10) RankInv Database*”) (Freeman et al., 2011).

We used quantitative RT-PCR (qPCR) analysis to confirm a difference in transcript levels during retinal development (Figure 5D). A two-way ANOVA revealed a main effect of strain, where *Xkr8* was significantly increased in B6/J compared to A/J (*p* = 0.02). We did not, however, observe a significant effect of age (*p* = 0.07) nor any significant interaction between age and strain (*p* = 0.08), although the difference between the strains appeared greatest at P5 (Figure 5D).

**FIGURE 6:**
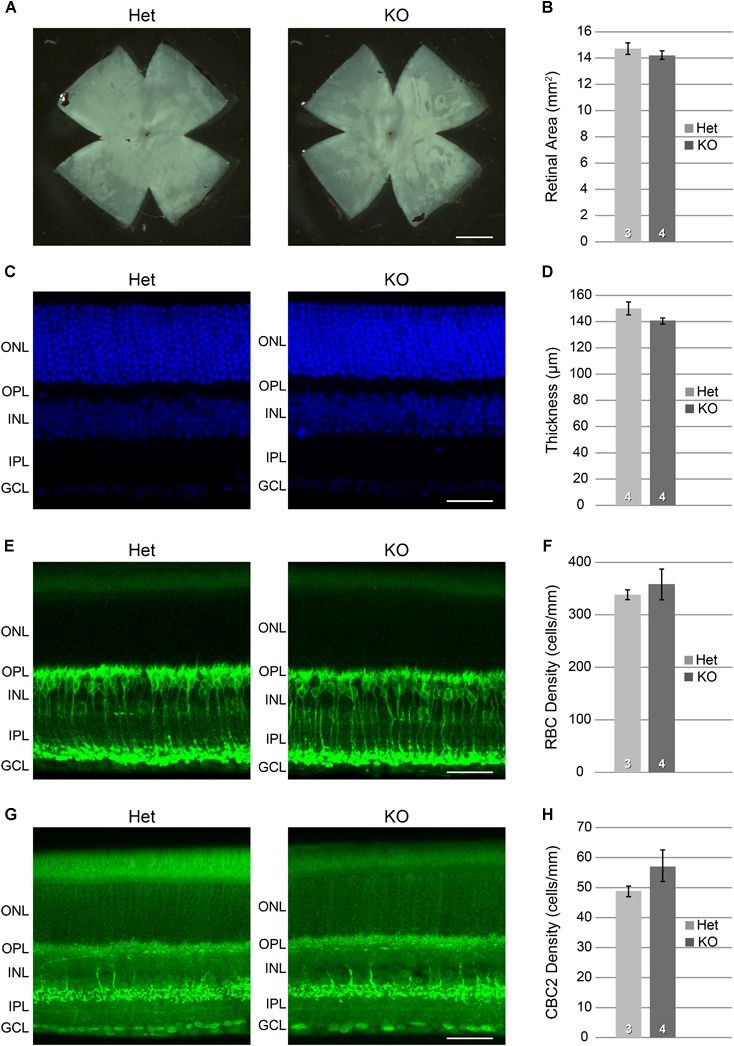
*Xkr8* KO versus heterozygous (Het) mice showed no difference in their total retinal area **(A,B)**, nor in the total thickness of the retina **(C,D)**. Individual retinal layers also did not differ (not shown). The densities of RBCs **(E,F)** and CBC2s **(G,H)** were slightly, though not significantly greater in the KO retinas (*p* > 0.1). n = the number of retinas examined in each condition. Scale bar = 1 mm for **(A)**; = 100 μm for **(C,E,G)**.

The above qPCR data, showing differential expression during the period of bipolar cell differentiation, are consistent with a role for *Xkr8* in the modulation of bipolar cell number in the mouse retina. To address this further, we examined *Xkr8* KO mice and compared them to heterozygous (Het) mice, which had previously been shown to retain normal XKR8 function (Suzuki et al., 2013). The eyes of KO mice appeared normal in size when compared to these control mice, and retinal area was unaffected (Figures 6A,B; *p =* 0.43), and nor was the retinal architecture, including total retinal thickness (Figures 6C,D; *p =* 0.13), or the thickness of individual retinal layers (not shown). To assess whether RBC and/or CBC2 number was altered in the absence of *Xkr8*, we determined the density of PKC-positive RBCs and SYT2-positive CBC2s in sections of retina from *Xkr8* KO and heterozygous littermate mice. While both RBC density and CBC2 density showed slight increases in the KO retinas, neither was statistically significant (*p =* 0.30 and 0.13, respectively) (Figures 6E–H).

While the above data suggest that complete removal *Xkr8* had minimal effect upon these two bipolar cell populations, this might reflect redundancy with other *Xkr* family members, such as *Xkr4* and *Xkr9*, which have been shown to have a similar function and share common genetic binding motifs, and *Xkr4* has been shown to be expressed in the eye (Suzuki et al., 2014). We therefore sought to directly modulate *Xkr8* expression as an alternative approach to circumvent this potential redundancy. We directly modulated *Xkr8* expression via plasmid electroporation, in an attempt to uncover a specific effect upon the population of bipolar cells. We electroporated either control or *Xkr8*-encoding plasmids driven by a ubiquitous CAG promoter, each including an IRES-*GFP* construct in order to report the efficiency of transfection. Electroporations were conducted on the day after birth (P2), during the period when bipolar populations continue to be generated (Morrow et al., 2008), as well as rod photoreceptors, Müller glia, and a few remaining amacrine cells (Young, 1985; Voinescu et al., 2010). Other studies using antibodies to PKC confirm we do in fact target bipolar cells amongst the population of transfected cells in these studies (Kautzman et al., 2018), but as the RBC and even moreso the CBC2 populations comprise a small proportion of the total number of bipolar cells, and because of the variable electroporation efficiency between individual mice, we simply quantified the change in the proportion of all GFP-positive cells situated in the outer half of the INL, being the bipolar cell stratum (where the majority of GFP-positive cells in the INL were situated) to that of all GFP-positive cells present.

**FIGURE 7:**
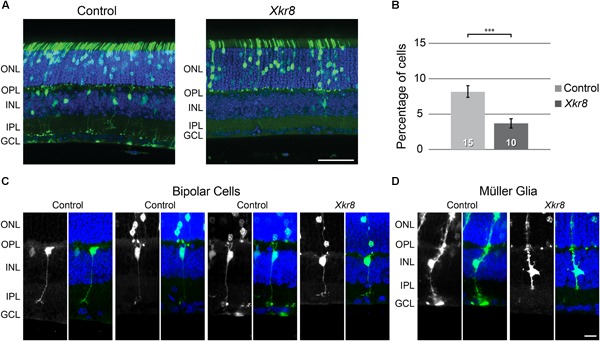
**(A)** Electroporation of control plasmids on P2 yielded GFP-positive cells occupying the ONL and INL, consistent with the postnatal genesis of rod photoreceptor, bipolar, Müller glial, and amacrine cell populations. **(B)** We quantified all GFP-positive cells in the section and determined the proportion situated in the outer half of the INL, where bipolar cell populations are resident. Electroporation of *Xkr8* encoding plasmids produced a reduction in the frequency of GFP-positive cells in the outer INL. The frequency of GFP-positive cells in this bipolar cell stratum is halved relative to the control plasmid condition (^∗∗∗^*p* < 0.001). n = the number of retinas examined in each condition. The labeled cells were commonly identifiable by their morphology, with bipolar cells being found in the outer half of the INL **(C)**. Muller glia, being positioned at a mid-depth in the INL **(D)**, were usually slightly more vitreal in their positioning in the INL, and would therefore not be included in counts of cells situated in the bipolar cell stratum. Scale bar = 50 μm for **(A)**; = 10 μm for **(C,D)**.

Sections of retinas from mice electroporated using *Xkr8*-encoding plasmids showed a conspicuous reduction in the frequency of such GFP-positive cells in the outer half of the INL relative to retinas from mice electroporated using control plasmids (Figure 7A). We sampled an average of 11 sections from each of the 25 electroporated retinas, yielding an average of 566 GFP-positive cells per retina. A determination of the positioning of those GFP-positive cells from the 15 control retinas and 10 *Xkr8* expressing retinas confirmed a significant decrease in the proportion of GFP-expressing cells in the outer INL, i.e., bipolar cells (Figure 7B; *p =* 0.0004). Most of the labeled cells in the outer half of the INL showed hallmarks of a bipolar cell’s morphology, with dendrites extending into the OPL and/or an axon terminal reaching to a particular depth in the IPL (Figure 7C). The only other cell type that was occasionally included in the population of GFP-positive cells in the outer half of the INL was the Müller glial cell, recognized by its irregular somal shape and/or processes extending to the outer or inner limiting membranes (Figure 7D). The present results, demonstrating that electroporation of *Xkr8* alters the relative number of bipolar cells in the transfected population, are consistent with the hypothesis that a *cis*-acting sequence variant modulates transcript levels of *Xkr8*, contributing to the variation in RBC and CBC2 number.

**FIGURE 8:**
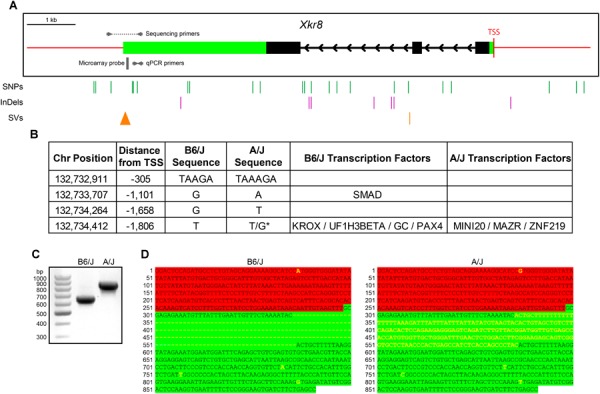
**(A)** The *Xkr8* gene has three exons (black boxes) and is ∼7,600 bp in size. 3^′^ and 5^′^ UTRs are denoted with green boxes and red lines indicate upstream and downstream regions. A/J and B6/J strains have multiple SNPs, InDels, and structural variants (SVs) within potential regulatory regions that discriminate their genomes (vertical lines). The 3^′^ UTR SV is depicted as a triangle to indicate the relative size of the insertion. **(B)** Of the four upstream sequence variants, two of them establish binding sites for various transcription factors in one or the other sequence. **(C,D)** Amplification of the 3^′^ end of *Xkr8*, using the sequencing primers indicated in **(A)**, confirms a 201 bp insertion in the A/J genome **(C)**, and sequencing of these amplicons localizes the structural variant to the 3^′^ UTR of the gene **(D)**. Green and red highlighting indicate the 3^′^ UTR and downstream regions, respectively, while the yellow lettering indicates variants between the strains. TSS, transcriptional start site. ^∗^Heterozygous variant.

*Xkr8* does not contain any variants within the coding sequence that discriminate the two parental genomes, but has many potential regulatory variants, including eight in the 3^′^ UTR, four upstream, four downstream, and 16 in intronic regions (Figure 8A). In addition, one InDel is located ∼300 base pairs (bp) upstream from the transcriptional start site, within the presumed promoter region. Of these four variants upstream of the TSS, two are predicted to render a transcription factor binding site discriminating the genomes (Figure 8B). Of particular note, the Mouse Genomes Project also predicted the presence of a large structural variant near the 3^′^ UTR region. Amplification of this region in the B6/J and A/J strains confirmed the existence of this structural variant, being an insertion in the A/J genome (Figure 8C). Sanger sequencing revealed that the 201 bp insertion is found wholly within the 3^′^ UTR (Figure 8D), indicating that this variant substantially alters the length of this region in the A/J strain, potentially disrupting levels of *Xkr8* transcripts through the creation or elimination of novel miRNA binding sites or by altering the secondary structure of the mRNA (Barrett et al., 2012; Tushev et al., 2018). One or more of these sequence variants, therefore, may be implicated in the differential expression of *Xkr8* across strains, ultimately affecting the numbers of RBCs and CBC2s observed in maturity.

## Discussion

The present study has shown that variation in the numbers of two different bipolar cell types each map to an identical genomic locus on Chr 4, where the mapped QTL accounts for a substantial portion of the overall variation in cell number. At this locus, we identified *Xkr8* as a particularly promising candidate gene, because *Xkr8* expression levels negatively correlate with bipolar cell numbers across a panel of RI strains, and because *Xkr8* expression is differentially regulated between the parental strains during retinal development. Variation in the expression of *Xkr8* mapped to the very locus where *Xkr8* is located, indicating *cis*-regulatory control of its expression, where strains with the *B* variant had higher levels of expression. We predicted that increasing expression of *Xkr8* should therefore negatively impact bipolar cell numbers, and confirmed this by altering *Xkr8* expression levels via plasmid electroporation. We consider in further detail below the role and specificity of *Xkr8* in modulating bipolar cell number.

Bipolar cells are believed to be overproduced during development, as their numbers are increased in the both *Bcl2*-overexpressing retinas as well as in *Bax*-deficient retinas (Strettoi and Volpini, 2002; Keeley et al., 2014a), while the frequency of apoptotic cells in the developing bipolar cell division of the inner nuclear layer is pronounced around P10 in the mouse retina (Young, 1984), when bipolar cells begin differentiating their processes and engaging in synaptogenesis (Morgan et al., 2006; Dunn et al., 2013). Modulating the frequency of naturally occurring cell death, then, would appear to be one means by which the final numbers of different types of bipolar cells are established during development. We paid particular attention to genes at the mapped interval on Chr 4 that have been shown to participate in regulating programmed cell death, where the candidate *Xkr8* stood out.

XKR8 is known to play a role in apoptosis by causing cells to expose phosphatidylserines on their surface to promote phagocytosis, specifically, by scrambling phospholipids (Suzuki et al., 2016). XKR8 is a large transmembrane protein that is activated following cleavage at a conserved caspase cleavage site near the C-terminal end of the protein via caspase-3 or caspase-7. Once cleaved, XKR8 then mediates phospholipid scrambling to expose phosphatidylserines on the cell surface that ultimately lead to engulfment (Suzuki et al., 2013, 2014). Given that *Xkr8* expression varies between the two parental strains during development (Figure 5D), transcript abundance of *Xkr8* may modulate the frequency of apoptosis during development, yielding the negative correlation between transcript levels and bipolar cell number observed in maturity across the RI strains (Figures 5A,B), and reduce the number of bipolar cells amongst the transfected population following electroporation of *Xkr8*-encoding plasmids during development (Figures 7B,C). Taken together, these data support the notion that that *Xkr8* plays a role in the specification of bipolar cell number by modulating apoptotic events during the time course of normal retinal development.

Curiously, *Xkr8* KO retinas did not yield any effect upon RBC or CBC2 numbers relative to Het control eyes (Figures 6F,H). This however may not be so surprising given that other *Xkr* family members are known to share biological functions as well as structural homology with *Xkr8*. For example, *Xkr4* and *Xkr9*, like *Xkr8*, each possess a caspase recognition site in their C-terminal region, and have been shown to substitute for *Xkr8* *in vitro* in promoting engulfment by macrophages (Suzuki et al., 2014). There may therefore be functional redundancy with other *Xkr* family members *in vivo* in the complete absence of *Xkr8*, yet this remains to be determined.

XKR8 function is antagonized by that of the flippase ATP11C (Segawa et al., 2014). ATP11C serves to direct phosphatidylserine from the outer to inner membrane surface. It contains the same caspase cleavage site as XKR8 so that following caspase cleavage, XKR8 becomes activated while ATP11C is inactivated. Data from the present study suggest that XKR8, perhaps in conjunction with ATP11C protein levels, may be tightly controlled to modulate caspase-mediated apoptosis during development. Gene variants that modulate *Xkr8* expression may therefore titrate the contributions of XKR8 in the presence of other factors modulating cell death pathways that ultimately affect total cell number. As cleavage of caspase-3 is no longer necessarily considered to be a “point-of-no-return” (Ding et al., 2016; Sun et al., 2017), one may envision a process by which the degree of *Xkr8* expression contributes to the balance between apoptosis or survival during this otherwise late stage in the cell death pathway.

As *in situ* hybridization for *Xkr8* in maturity showed widespread expression across the retina, the role of *Xkr8* in modulating bipolar cell number might not necessarily be restricted to modulating the number of only the RBCs and CBC2s, even if the numbers of Type 3b and Type 4 CBCs also quantified in the RI strains did not map to this same genomic locus. For those cell types, other selective genetic variants may play outsized roles relative to *Xkr8*, preventing *Xkr8* from revealing itself across the RI strains in the presence of those other stronger contributing factors. *Xkr8* may also play other distinct roles besides modulating programmed cell death. For instance, the conspicuous expression in the mature ONL may be associated with a role in the phagocytosis of outer segment debris during the normal process of disk shedding triggered by phosphatidylserine exposure (Ruggiero et al., 2012). *Xkr8* has also been shown to regulate myocyte terminal differentiation when overexpressed, and this process was independent of caspase cleavage (Kim et al., 2017). While the precise mechanism remains to be determined, the present results implicate *Xkr8* as participating in the control of bipolar cell number during retinal development.

## Author Contributions

AK conducted the KO analyses. AK and PK conducted the qPCR, electroporations, and analysis of *Xkr8* gene variants. PK conducted the *in situ* hybridization and XKR8 immunofluorescence. CA analyzed the electroporated retinas. SL counted CBC2 cells in the RI strains. IW and PK conducted the QTL mapping. AK and IW conducted the candidate gene analysis. AK and BR wrote the manuscript.

## Conflict of Interest Statement

The authors declare that the research was conducted in the absence of any commercial or financial relationships that could be construed as a potential conflict of interest.

## Footnotes

1 www.genenetwork.org

2 https://fiji.sc/
